# Effects of the Supercritical Fluid Extract of *Magnolia figo* on Inducing the Apoptosis of Human Non-Small-Cell Lung Cancer Cells

**DOI:** 10.3390/molecules28217445

**Published:** 2023-11-06

**Authors:** Chun-Sheng Kuo, Shih-Yun Chen, Jen-Chieh Tsai

**Affiliations:** 1 Fethiann Molecule Applied Co., Ltd., Yilan 260011, Taiwan; frederickuo@fethiann.tw; 2 Department of Medicinal Botanicals and Foods on Health Applications, Da-Yeh University, Changhua 515006, Taiwan; lulu53190225@gmail.com

**Keywords:** *Magnolia figo*, FMO, lung cancer, A549 cell, apoptosis, p53/Bcl-2/Bax

## Abstract

Lung cancer has a high incidence rate worldwide, necessitating the development of new drugs. Although *Magnolia figo* (Lour.) DC. is known for its medicinal properties, studies on its efficacy against lung cancer are lacking. This study investigated whether the supercritical fluid extract of *M. figo* (FMO) can induce apoptosis in A549, a human non-small-cell lung cancer cell line. The cell viability was assessed using an MTT assay. A terminal deoxynucleotidyl transferase dUTP nick end labeling (TUNEL) analysis and flow cytometry analysis were conducted. The expression of factors was assessed through Western blotting analyses. Gas chromatography–mass spectrometry (GC-MS) was performed. The results revealed that FMO treatment exhibited cytotoxicity, demonstrating dose-dependent effects. The TUNEL analysis and flow cytometry analysis revealed that FMO induced apoptosis in A549 cells. The Western blotting analysis revealed that FMO upregulated the expression of p53 and Bax protein, and downregulated the expression of Bcl-2 protein. The GC-MS analysis revealed eight components identified in FMO. These findings indicate that FMO can induce A549 apoptosis through the p53/Bcl-2/Bax pathways, confirming the apoptotic effects of *M. figo* on lung cancer cells. These results highlight the potential, for the first time, of *M. figo* as a source for developing novel drugs for lung cancer treatment.

## 1. Introduction

Lung cancer is one of the leading causes of cancer-related deaths, ranking third and second in terms of incidence rate and mortality rate worldwide, respectively [1]. Non-small-cell lung cancer (NSCLC) is the most common type of lung cancer [2], accounting for 85% of all lung cancer cases globally [3]. The current treatment options for NSCLC mainly include surgery, chemotherapy, and targeted therapy. However, these treatment methods have limitations, necessitating studies that aim to develop new cancer drugs that can increase lung cancer cell apoptosis and lower patient mortality rates. Given the antioxidative, anti-inflammatory, and anticancer properties of many natural products, exploring plants and their active components for potential anticancer effects is a noteworthy endeavor.

In organisms, apoptosis is regulated through two main pathways: the extrinsic pathway and the intrinsic pathway [4]. When cells are stimulated and receive death signals, they activate the p53 protein and downstream caspase, resulting in apoptosis [5]. Studies have demonstrated that the p53 protein can increase the permeability of mitochondrial membranes, thereby inducing cell death [6]. In contrast, the Bcl-2 (B-cell lymphoma-2) protein family, which consists of proapoptotic and antiapoptotic proteins, plays a crucial role in the regulation of mitochondrial integrity [7]. These proteins are primarily present in mitochondrial membranes, with the proapoptotic protein members (e.g., Bax (Bcl-2-associated X)) usually found in the cytoplasm. When Bax receives apoptotic signals, it translocates to the surface of the mitochondria, creating holes in the mitochondrial membrane, which results in a decrease in the membrane’s electric potential and an increase in its permeability. These changes trigger the release of apoptosis factors. For example, cytochrome c, when released into the cytoplasm, activates caspase 9 and triggers the caspase-3 cascade reaction, thereby inducing apoptosis [5,8].

*Magnolia figo* (Lour.) DC., a plant from the family Magnoliaceae, is native to Fujian and Guangdong in southern China. It is now also found in tropical and subtropical regions, such as Taiwan, eastern India, southern China, Malaysia, and Indonesia. *M. figo* has a distinct, strong banana-like aroma and has many uses, including ornamental purposes and as an ingredient in scented tea. *M. figo* is mild in nature, and bitter and astringent to the taste. Its buds and leaves can be used as medicine. The buds of the plant are highly aromatic and can be used for eliminating body moisture, promoting *qi* circulation, removing obstructions, and stimulating menstrual flow. Therefore, *M. figo* buds have been increasingly used to treat irregular menstruation, dysmenorrhea, *qi* stagnation, abdominal distension, and rhinitis. The leaves of *M. figo* exhibit anti-inflammatory and detoxification properties, and can be used to treat bruises, sore throats, and bronchitis [9]. Studies have demonstrated that *M. figo* has antioxidative and anti-inflammatory effects. In traditional Indian medicine, *M. figo* is known for its potential to treat high blood pressure [10].

Green extraction methods have environmentally friendly characteristics, such as supercritical carbon dioxide extraction, supercritical fluid extraction, and aqueous two-phase extraction, which are energy-saving and environmentally friendly extraction methods [11,12,13]. An aqueous two-phase system is a liquid–liquid extraction method in which both phases are formed mainly by water [14]. Supercritical fluid extraction uses supercritical fluids as extraction solvents, and is a suitable technique for extracting and purifying various compounds. Carbon dioxide (CO_2_) is the most used supercritical fluid [15]. In this study, supercritical carbon dioxide extraction was utilized to obtain the supercritical fluid extract of *M. figo* (FMO). A549 cells are an NSCLC cell line in humans. A549 cells in lung tissue are squamous, long, and narrow, and are responsible for diffusing substances, such as water and electrolytes, in the pulmonary alveoli. Additionally, they have the ability to synthesize lecithin containing highly unsaturated fatty acids through the citicoline pathway. A549 cells are widely used as a model for studying lung cancer and for developing new drugs [16]. Additionally, they have been used as a model for type II pulmonary alveolar epithelial cells, and are practical for studying metabolic processes in lung tissue and the possible mechanisms underlying drug delivery to tissue [17]. Studies investigating the effects of *M. figo* on NSCLC and apoptosis induction are lacking. Therefore, the present study used the A549 cell model to evaluate the effects of *M. figo* extract, specifically the FMO extract, on promoting apoptosis in A549 lung cancer cells, and to investigate whether FMO facilitated apoptosis through the p53/Bcl-2/Bax pathways.

## 2. Results

### 2.1. Effects of FMO on A549 Cell Viability

**Figure 1:**
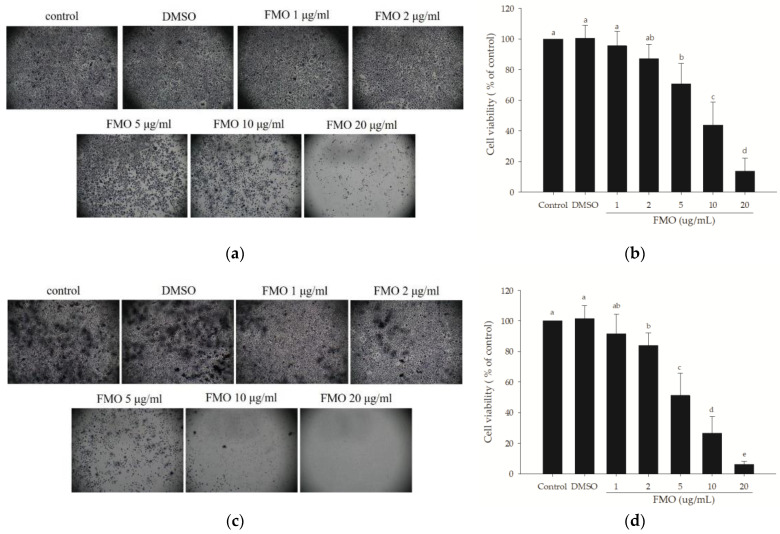
Effects of different FMO concentrations on A549 cell viability at 24 h (**a**,**b**) and 48 h (**c**,**d**). The purple formazan crystals in the figure denote the number of living cells. The results are presented as mean ± SD (*n* = 3), and the different letters indicate significant differences (*p* < 0.05).

MTT was used to measure the viability of cells on stimulation. The results (Figure 1) reveal that the addition of 0.1% DMSO did not exhibit any cytotoxicity at 24 or 48 h. When A549 cells were treated with different concentrations of FMO (i.e., 1, 2, 5, 10, and 20 μg/mL), after 24 h of treatment, the cell viability of the 5, 10, and 20 μg/mL FMO were found to be significantly lower than that of the control group (*p* < 0.05), with the half-maximal inhibitory concentration (IC_50_) of the cells determined to be 8.4 ± 2.8 μg/mL (Figure 1a,b). After 48 h of treatment, the cell viability of the 2, 5, 10, and 20 μg/mL FMO were significantly lower than that of the control group (*p* < 0.05), with an IC_50_ of 5.1 ± 1.3 μg/mL (Figure 1c,d). Thus, a dose-dependent response to FMO treatment was observed for both treatment durations. However, an FMO dose of ≥5 μg/mL resulted in cell death. Therefore, FMO concentrations of 1, 5, and 10 μg/mL were chosen for subsequent experiments.

### 2.2. TUNEL Staining Revealed Apoptosis in A549 Cells Induced by FMO

**Figure 2:**
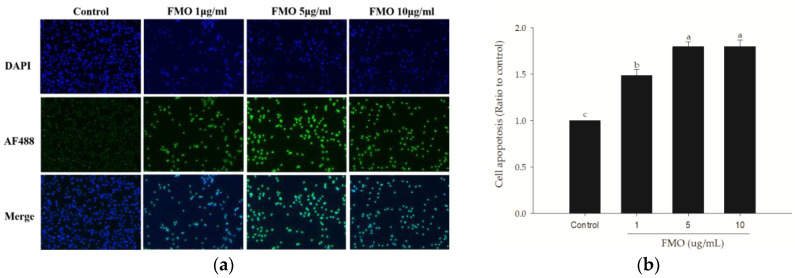
TUNEL fluorescence analysis of the effects of a 24 h treatment with different FMO concentrations on A549 apoptosis. (**a**) Representative TUNEL staining for A549 cells. The blue fluorescence represents the nuclei stained with DAPI, whereas the green fluorescence (AF488) indicates the presence of apoptotic cells. Images were captured using a fluorescent microscope with a 40× objective lens, and ZEN software version 13 was used for image processing. (**b**) Quantitative analysis of TUNEL. The results are presented as mean ± SD (*n* = 3), and the different letters indicate significant differences (*p* < 0.05).

In Figure 2, the areas stained with DAPI represent the cell nuclei, and the cells stained with AF488 indicate that they are apoptotic. Compared to the control group, the groups treated with 1, 5, and 10 μg/mL FMO exhibited a greater proportion of green fluorescence, indicating that FMO treatment for 24 h induced apoptosis in the A549 cells.

### 2.3. Annexin V Assay Revealed FMO-Induced Apoptosis in A549 Cells

**Figure 3:**
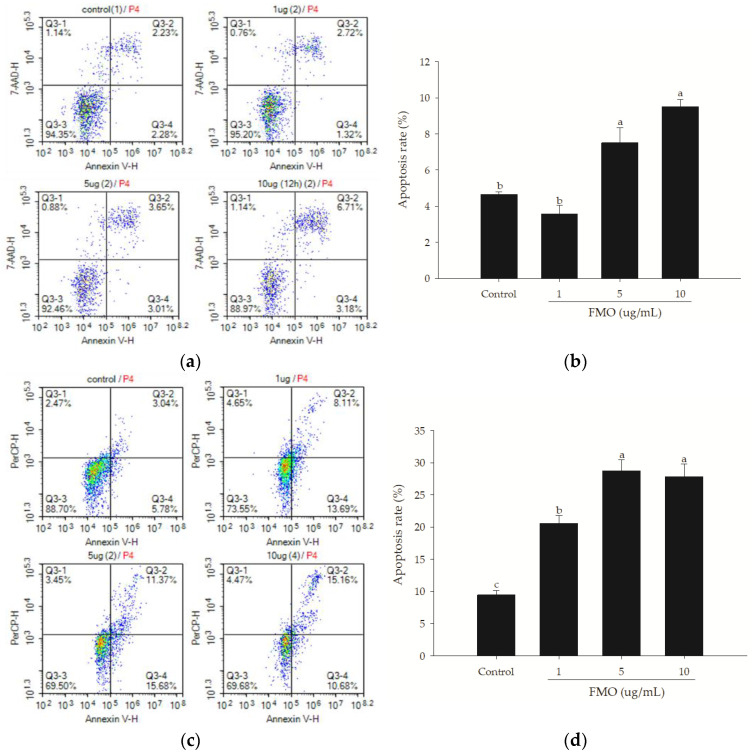
Flow cytometry analysis of the effects of different FMO concentrations on A549 apoptosis at 12 (**a**,**b**) and 24 h (**c**,**d**). (**a**,**c**) were density plots for cell apoptosis analysis. P4, labeled in red, indicates the selected region for analysis. Annexin V/PI stain was used to assess apoptosis. The upper-left quadrant represents necrotic cells, the lower-left quadrant represents living cells, the upper-right quadrant represents late-stage apoptotic cells, and the lower-right quadrant represents early-stage apoptotic cells. The results are presented as mean ± SD (*n* = 3), and the different letters indicate significant differences (*p* < 0.05).

To examine whether FMO induces lung cancer cell apoptosis, Annexin V-FITC/7-AAD staining was employed to harvest the cells, and flow cytometry was used to analyze the effects of FMO on A549 apoptosis. Figure 3 presents the effects of different FMO concentrations on A549 apoptosis after treatment for 12 (Figure 3a,b) and 24 h (Figure 3c,d). The flow cytometry results are presented in four quadrants: the upper-right quadrant presents the late-stage apoptotic cells, the lower-right quadrant presents the early-stage apoptotic cells, the upper-left quadrant presents the necrotic cells, and the lower-left quadrant presents the living cells. After a 12 h treatment, the 5 and 10 μg/mL FMO groups exhibited apoptosis. Furthermore, after a 24 h treatment, the 1 μg/mL FMO group also exhibited apoptosis. These results indicate that FMO induced apoptosis in the A549 cells.

### 2.4. Effects of FMO on A549 Cell Apoptosis-Related Proteins

**Figure 4:**
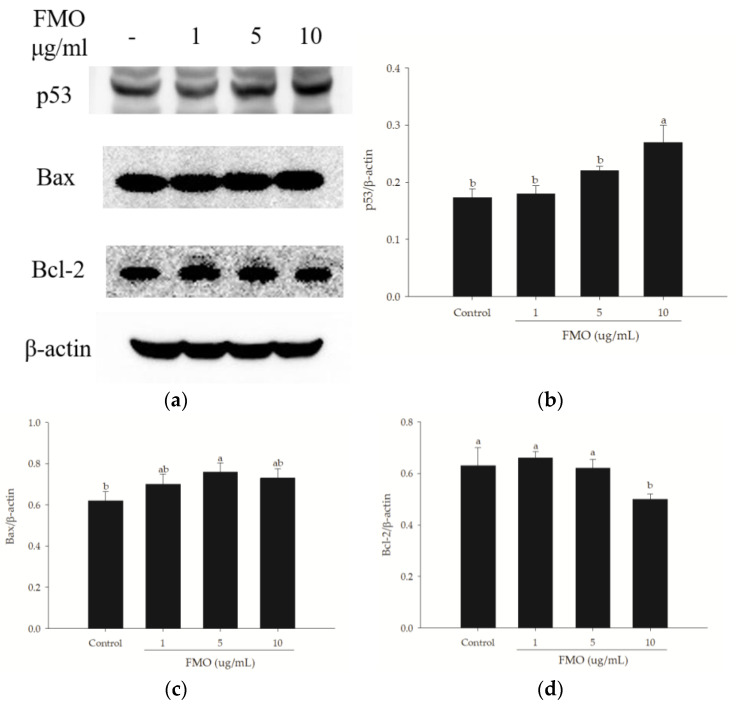
Protein expression of p53, Bax, and Bcl-2 in A549 cells treated with different concentrations of FMO. (**a**) Western blotting analysis for p53, Bax, and Bcl-2 in A549 cells. Β-actin was used as a control; (**b**–**d**) Quantitative analyses of p53 (**b**), Bax (**c**), and Bcl-2 (**d**) expressions in comparison to β-actin. The results are presented as mean ± SD (*n* = 3). The letter a or b indicates significant differences (*p* < 0.05), whereas ab in combination indicates nonsignificant differences.

To elucidate the molecular mechanisms underlying FMO-induced A549 apoptosis, the expression of apoptosis-related proteins was evaluated using Western blotting. As shown in Figure 4, treatment with 5 and 10 μg/mL FMO led to an increase in p53 and Bax protein expression. Moreover, a dose-dependent relationship was observed, with higher FMO doses corresponding to increased protein expression. Conversely, the protein expression of Bcl-2 was significantly reduced. These results indicate that FMO can activate p53 and Bax signaling pathways, thereby promoting A549 apoptosis.

### 2.5. FMO GC-MS Composition Analysis

**Figure 5:**
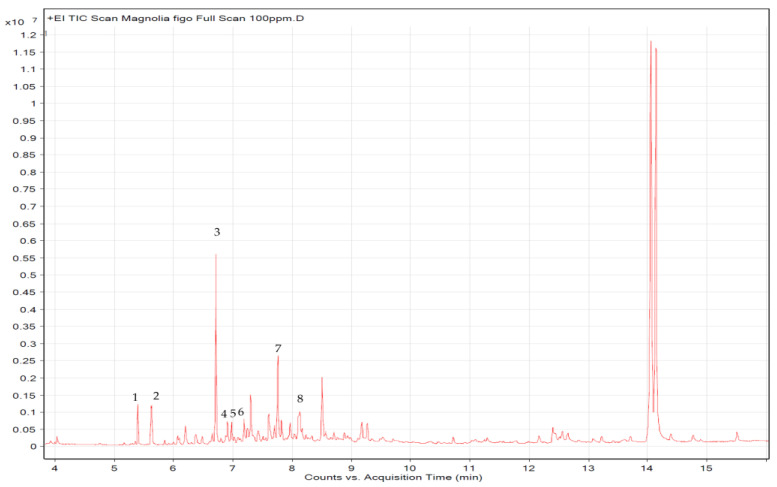
The GC-MS diagram of FMO composition. 1: β-Elemene; 2: γ-Elemene; 3: Caryophyllene Oxide; 4: (1R,3E,7E,11R)-1,5,5,8-Tetramethyl-12-oxabicyclo[9.1.0]dodeca-3,7-diene; 5: cis-α-Copaene-8-ol; 6: Spathulenol; 7: Ledene Oxide (II); 8: Ledene Alcohol.

**Figure 6:**
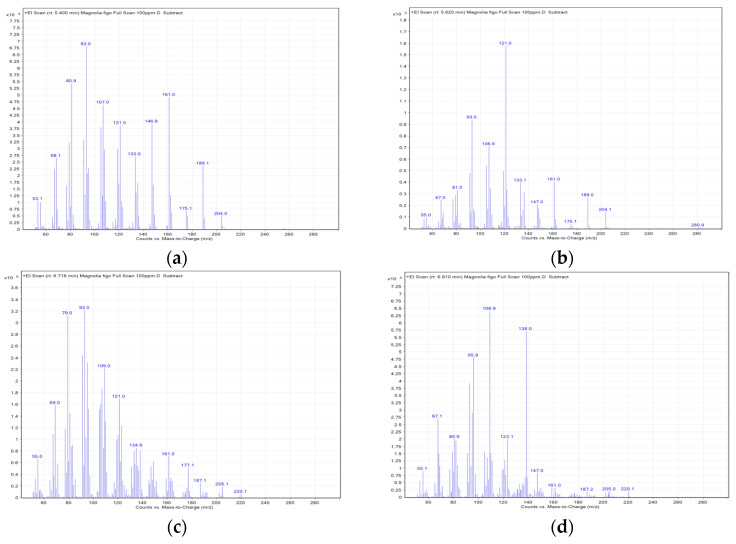

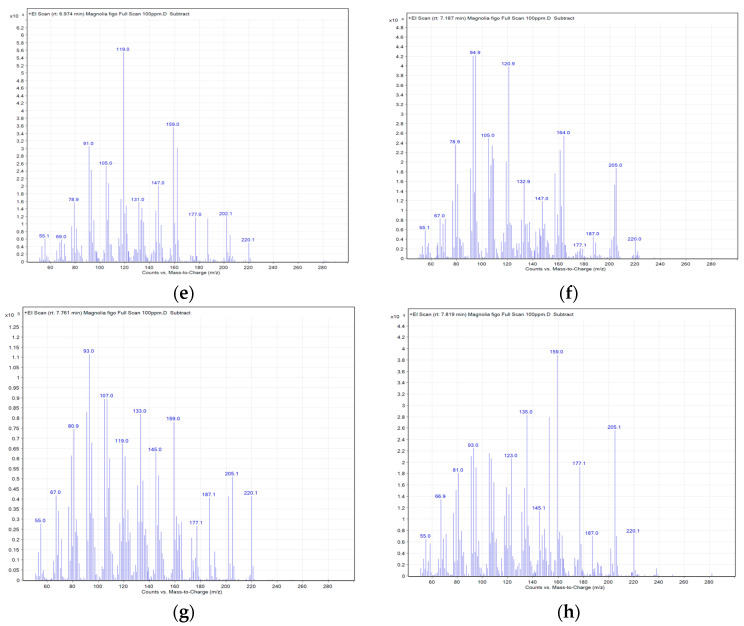
The MS spectra of FMO composition: (**a**) β-elemene; (**b**) γ-elemene (**c**) caryophyllene oxide; (**d**) (1R,3E,7E,11R)-1,5,5,8-Tetramethyl-12-oxabicyclo[9.1.0]dodeca-3,7-diene; (**e**) cis-α-Copaene-8-ol; (**f**) spathulenol; (**g**) ledene oxide (II); (**h**) ledene alcohol.

The FMO composition was analyzed through GC-MS, and the results are presented in Figure 5 and Figure 6 and Table 1. A total of eight FMO components were identified, containing β-elemene, γ-elemene, caryophyllene oxide, (1R,3E,7E,11R)-1,5,5,8-Tetramethyl-12-oxabicyclo [9.1.0]dodeca-3,7-diene, cis-α-Copaene-8-ol, spathulenol, ledene oxide (II), and ledene alcohol.

## 3. Discussion

Apoptosis, a process of programmed cell death, is induced through various pathways. It plays a crucial role in multiple physiological processes, such as growth, immune system regulation, tissue repair, and control of tumor cell growth. Inducing cancer cell apoptosis through drugs is an effective anticancer strategy [18,19]. In recent years, natural products have been increasingly used for the development of new anticancer drugs. In this study, the effects of FMO on A549 cell viability were investigated, and the results reveal a substantial decrease in A549 cell survival rates, indicating the apoptotic effects of FMO on NSCLC cells. Apoptosis is marked by various morphological changes, such as pyknosis, cell surface changes, and chromosome condensation [18]. To assess the apoptotic effects of FMO on the A549 cells, TUNEL analysis and flow cytometry were used in this study. The results indicate a significant increase in the percentage of apoptotic cells treated with FMO in a concentration-dependent manner, confirming the ability of FMO to induce apoptosis in A549 cells.

p53, Bax, and Bcl-2 are crucial regulatory proteins involved in apoptosis. p53 is a transcription factor and plays an important role in the regulation of the cell cycle, which can be activated in response to DNA damage and other stressful situations [8,20]. Upon activation, p53 triggers the transcription of target genes, including Bax and Bcl-2, thereby inducing apoptosis. Bax and Bcl-2 are members of the Bcl-2 family of proteins and play critical roles in the apoptosis pathway [21]. Bax facilitates apoptosis and induces the release of cytoplasmic pigment c in the apoptosis pathway, triggering apoptosis. In contrast, Bcl-2 is an antiapoptotic protein that inhibits Bax activity, thereby delaying apoptosis [22]. Thus, in the apoptosis pathway, p53 mainly regulates the expression of genes, such as Bax and Bcl-2, thereby influencing the apoptosis process. p53 may also trigger apoptosis by inducing Bax expression and reducing Bcl-2 expression [21]. In this study, the expression levels of p53, Bax, and Bcl-2 were evaluated in response to FMO treatment. The results reveal an increase in p53 protein expression upon FMO treatment. Accordingly, FMO may activate p53, leading to its translocation from the mitochondria, and subsequent interaction with apoptotic proteins. This interaction may increase the permeability of the outer mitochondrial membranes, which triggers the release of apoptotic factors in response, thereby inducing apoptosis. Additionally, FMO reduced the expression of antiapoptotic Bcl-2 proteins and increased the expression of proapoptotic Bax proteins. The Bax/Bcl-2 ratio is crucial for regulating apoptosis in lung cancer cells and disrupting mitochondrial homeostasis. These effects of FMO on p53, Bax, and Bcl-2 indicate its potential to promote apoptosis in lung cancer cells. Therefore, we suggest that FMO may induce apoptosis in A549 cells by affecting the p53/Bcl-2/Bax pathway.

The GC-MS analysis reveals that the main FMO components are β-elemene, γ-elemene, caryophyllene oxide, (1R,3E,7E,11R)-1,5,5,8-Tetramethyl-12-oxabicyclo[9.1.0]dodeca-3,7-diene, cis-α-Copaene-8-ol, spathulenol, ledene oxide (II), and ledene alcohol. Among the elemene components, β-elemene has the strongest anticancer activity; γ-elemene and δ-elemene are considered auxiliary components that are capable of synergistically enhancing the anticancer effects of β-elemene. β-elemene has been shown to inhibit tumor cell migration and adverse reactions, and is widely used in the treatment of various cancers [23,24]. β-elemene has also been found to induce A549 apoptosis, reduce Bcl-2 expression, increase Bax expression, and cause PARP cleavage [25]. Moreover, it has demonstrated anti-inflammatory effects. In an LPS-induced RAW264.7 macrophage inflammation model, β-elemene strongly inhibited proinflammatory cytokines (e.g., IL-6, TNF-α, and IL-1β), and iNOS and IL-10 [26]. Caryophyllene oxide, another active component of *M. figo,* exerts anti-inflammatory, anticancer, and weak antiviral effects [27,28]. It has been shown to induce NSCLC apoptosis and cause the arrest of the cell cycle, and is associated with an increase in the expression of caspase-3, caspase-7, caspase-9, and Bax, and a decrease in the expression of Bcl-2 [29]. Thus, we surmise that the apoptotic effects of FMO may be attributed to these main functional components.

## 4. Materials and Methods

### 4.1. FMO Extraction and Preparation Methods

*M. figo* is mainly grown in Yilan, Taiwan. In this study, we obtained *M. figo* samples from Fethiann Co., Ltd., Yilan, Taiwan. Supercritical extraction equipment (SE-2000-10000, TST Co., Ltd., Changhua, Taiwan) was used for the extraction process. Into the extraction tank, 1 kg of dried *M. figo* petals was placed. The extraction conditions included a pressure of 4000 PSI, a temperature of 40 °C, and an extraction time of 8 h. The process yielded *M. figo* extract, known as FMO, with an extraction rate of approximately 0.12%. In the cell experiments, 100 mg of FMO was dissolved in 1 mL of DMSO, and the solution was diluted to 100 μg/mL by using 1× PBS. To obtain low concentrations of FMO, the solution was further diluted using the culture medium.

### 4.2. Cell Culturing

The human NSCLC A549 cells used in this study were purchased from the Food Industry Research and Development Institute, Hsinchu, Taiwan. The A549 cells were cultured in a F-12 K medium (i.e., Kaighn’s Modification of Ham’s F-12 Medium), supplemented with 10% fetal bovine serum and 1% antibiotics. The cells were grown in an incubator with 5% CO_2_ and at a temperature of 37 °C. The experiment began once the cells reached a confluence of 80%.

### 4.3. Cell Viability Analysis

The A549 cells were seeded in a 96-well culture plate at a density of 1 × 10^6^/well. The plate was subsequently placed in an incubator with 5% CO_2_, with the temperature set to 37 °C, for 24 h. After the cells attached themselves to the bottom of the plate, different doses of the FMO samples were added to the respective wells, and the plate was incubated for 24 h. Next, 100 μL of 0.5 mg/mL MTT (3-(4,5-dimethylthiazol-2-yl)-2,5-diphenyltetrazolium bromide) solution was added to each well, and the plate was incubated in the dark for 4 h. Thereafter, the old solutions were removed, and 100 μL of isopropanol was added to each well. The absorbance of the resulting solution was measured at a wavelength of 562 nm by using an enzyme-linked immunosorbent assay reader (brand). To calculate cell viability, the experimental group/control group data were multiplied by 100% according to the formula. Each experiment was repeated 3 times.

### 4.4. Annexin V Apoptosis Assay (Cell Surface Phosphatidylserine Analysis)

The A549 cells were seeded in a 6 cm well plate (2 × 10^5^/well), and the plate was incubated with 5% CO_2_ at a temperature of 37 °C for 2 days to allow the cells to attach to the bottom of the plate. The cells were then treated with different doses of FMO (i.e., 0, 1, 5, and 10 μg/mL), and incubated with 5% CO_2_ at a temperature of 37 °C for 24 h. The cells were then harvested using the Annexin V-FITC/7-AAD Apoptosis Kit (Elabscience biotechnology Co. Ltd., Houston, TX, USA), and flow cytometry analysis was performed to evaluate the apoptosis levels in the treated cells. Each assay was repeated 3 times.

### 4.5. Terminal Deoxynucleotidyl Transferase dUTP Nick End Labeling Assay

A terminal deoxynucleotidyl transferase dUTP nick end labeling (TUNEL) assay was performed by placing sterilized, round glass slides into a 3 cm dish at a density of 2 × 10^5^/well. Next, the A549 cells were seeded onto the dish and incubated with 5% CO_2_ at a temperature of 37 °C for 2 days to allow the cells to attach to the bottom of the dish. After the cells had attached to the dish, different doses of the FMO samples were added, and the dish was incubated with 5% CO_2_ at a temperature of 37 °C for 24 h. Finally, the One-step TUNEL In Situ Apoptosis Kit (Elabscience biotechnology Co. Ltd., Houston, TX, USA) was employed according to the manufacturer’s instructions, and the cells were visualized using fluorescent microscopy.

### 4.6. Western Blotting

A Western blot analysis was performed to analyze the protein expression of p53, Bcl-2, and Bax in the A549 cells. The A549 cells were treated with a lysis buffer to obtain a cell supernatant, and a protein analysis reagent was used to analyze the protein content of the supernatant. Subsequently, the protein samples with the appropriate concentration were boiled for 5 min to allow for protein denaturation. According to the experimental requirements, a separating gel was prepared. Once the gel solidified, a stacking gel was prepared. A running buffer was poured into an electrophoresis tank, and an appropriate amount of protein samples was added to each well for protein separation. After the electrophoresis process was completed, the electrophoresis films were placed on filter papers that had been wetted with 1× transfer buffers. Polyvinylidene fluoride (PVDF) transfer membranes soaked in methanol were placed on the gel, and the wet filter papers were placed on top of the PVDF membranes. The membranes were folded and secured using clips, and were placed in a trans-blot cell. Trans-blotting was performed at 4 °C. During the blotting process, the PVDF membranes were washed with phosphate-buffered saline with tween (PBST) buffer for 10 min, and the PVDF membranes were soaked in T20 (PBS) blocking buffer at room temperature to prevent the nonspecific binding of antibodies to the PVDF membranes. Then, the PVDF membranes were incubated with PBST buffer containing an appropriate amount of antibodies, and shaken slowly at 4 °C overnight. On the following day, the PVDF membranes were rinsed 3 times (10 min each time) with PBST buffer, and then incubated with PBS buffer with an appropriate amount of secondary antibodies. Then, the PVDF membranes were allowed to stand at room temperature for 2 h with gentle shaking. At the end of the 2 h, the PVDF membranes were washed with the PBST buffer 3 times (10 min each time). Finally, the moisture on the PVDF membranes was dried, and an ECL detection kit was applied to the membranes. The PVDF membranes were then sealed in a plastic box, and imaging was performed using a luminescence imaging analysis system (brand).

### 4.7. Gas Chromatography/Mass Spectrometry Composition Analysis

The composition of the FMO was analyzed in this study through gas chromatography–mass spectrometry (GC-MS) (Agilent 7890 GC/7000C Triple Quadrupole, CA, USA). The separation column used in the GC was DB-5ms (30 m × 0.25 μm × 0.25 mm). The temperature-increase gradient for the GC-MS analysis was as follows: An initial analysis temperature of 60 °C was maintained for 1 min. The temperature was increased by 40 °C every minute. At 170 °C, the temperature was maintained for 0 min and then increased by 10 °C per minute to reach 310 °C. At 310 °C, the temperature was maintained for 2.25 min. The sample injection volume was 1.0 μL. The carrier gas was helium, the injection port temperature was 270 °C, the ion source temperature was 230 °C, and the column low rate was 1.7 mL/min. The instant splitless injection mode (Splitless Injection) was used. The mass spectrometer temperature was 150 °C. The sample weighed approximately 10 mg, and was placed into a 50 mL volumetric flask and dissolved using acetone. The concentration was 100 g/mL and was analyzed using GC/MS.

### 4.8. Statistical Analysis

Experimental data were analyzed using IBM SPSS (SPSS, Inc., Chicago, IL, USA). One-way ANOVA and Duncan’s new multiple range tests were employed to analyze whether significant differences existed between the data from the different groups. The data are expressed as mean ± SD, and a statistical significance was defined as *p* < 0.05.

## 5. Conclusions

**Figure 7:**
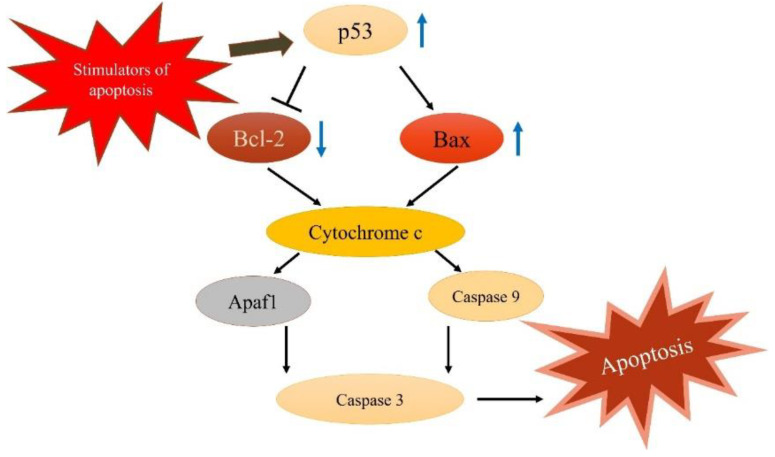
Apoptosis signaling pathway. Upon stimulation, the A549 cells activate p53, which translocates into the nuclei and triggers various reactions and cellular processes, including growth. This p53 activation leads to the upregulation of the proapoptotic protein Bax, and downregulation of the antiapoptotic protein Bcl-2. Subsequently, cytochrome c is released, which triggers caspase 3 production, finally leading to apoptosis.

The findings of this study strongly show that FMO has the ability to promote apoptosis in lung cancer cells. FMO treatment can upregulate the levels of p53 and Bax, while downregulating the expression of Bcl-2, thereby inducing apoptosis in A549 cells through the p53/Bcl-2/Bax signaling pathway (Figure 7). Furthermore, the GC-MS analyses identified the functional components of FMO, such as β-elemene and caryophyllene oxide. This study is the first to suggest the apoptotic potential of FMO in lung cancer cells. Although more in-depth research is required, these findings support the potential of FMO as a promising candidate for the development of lung cancer drugs.

## Figures and Tables

**Table 1:** FMO composition determined using GC/MS analysis.

No.	Retention Time	Formula	Type	Compound Name
1	5.400	C_15_H_24_	Sesquiterpenes	β-Elemene
2	5.620	C_15_H_24_	Sesquiterpenes	γ-Elemene
3	6.716	C_15_H_24_O	Sesquiterpenols	Caryophyllene Oxide
4	6.910	C_15_H_24_O	Sesquiterpenols	(1R,3E,7E,11R)-1,5,5,8-Tetramethyl-12-oxabicyclo[9.1.0]dodeca-3,7-diene
5	6.974	C_15_H_24_O	Sesquiterpenols	cis-α-Copaene-8-ol
6	7.187	C_15_H_24_O	Sesquiterpenols	Spathulenol
7	7.761	C_15_H_24_O	Sesquiterpenols	Ledene Oxide (II)
8	8.128	C_15_H_24_O	Sesquiterpenols	Ledene Alcohol

## Data Availability

The data presented in this study are available in the article.
